# Extraction and Bioactivity Analysis of Major Flavones Compounds from *Scutellaria baicalensis* Using In Vitro Assay and Online Screening HPLC-ABTS System

**DOI:** 10.1155/2014/563702

**Published:** 2014-09-01

**Authors:** Kwang Jin Lee, Pil Mun Jung, You-Chang Oh, Na-Young Song, Taesoo Kim, Jin Yeul Ma

**Affiliations:** KM-Based Herbal Drug Development Group, Korean Institute of Oriental Medicine (KIOM), 1672 Yuseongdae-ro, Yuseong-gu, Daejeon 305-811, Republic of Korea

## Abstract

The extraction efficiency of a number of solvent compositions for the improvement of bioactive compounds yield from *S. baicalensis* has been investigated. Also, free radical scavengers in the glycoside baicalin (BG), wogonoside (WG), aglycon baicalein (B), and wogonin (W) compounds of *S. baicalensis* were screened, identified, and quantified using coupled offline ABTS and online screening HPLC-ABTS assay. Increasing ethanol content fractions resulted in decreased extract yield of bioactive compounds. In this case, the best yield of 37.01 mg/g in BG, WG, B, and W compounds was obtained by a dipping method with an extraction time of 4 h. In addition, the yield (43.05%) and IC_50_ (34.04 *μ*g/mL) determined through ABTS assay of the 60% aqueous ethanol extract were the most satisfactory of all solvent solutions tested. This result shows that an online screening HPLC-ABTS assay can be a powerful technique for the rapid characterization of bioactivity compounds in plant extracts. Moreover, their anti-inflammatory activities were evaluated via analyzed inhibitory effect on NO and inflammatory cytokine production. Furthermore, WG and W exhibited the strong inhibitory effects on inflammatory mediator production including NO, IL-6, and IL-1*β* in LPS-stimulated RAW 264.7 macrophages.

## 1. Introduction

*Scutellaria baicalensis *is one of the most widely used medicinal herbs for the treatment of various inflammatory diseases, such as hepatitis, tumors, and diarrhea in East Asian countries [1, 2].* S. baicalensis *contains a variety of flavones, phenylethanoids, amino acids, sterols, and essential oils [3]. Its dried roots contain flavonoids such as baicalin, baicalein, wogonin, wogonin 7-*O*-glucuronide, oroxylin A, and oroxylin A 7-*O*-glucuronide [4]. These four major flavones: glycoside baicalin (BG, MW; 446.37, C_21_H_18_O_11_), wogonoside (WG, MW; 460.39, C_22_H_20_O_11_), aglycon baicalein (B, MW; 270.24, C_15_H_10_O_5_), and wogonin (W, MW; 284.27, C_16_H_12_O_5_) were reported to be the main bioactive components in* S. baicalensis *[5, 6]. Besides its anti-inflammation and anticancer properties,* S. baicalensis* is effective in treating bacterial and viral infections, reducing the total cholesterol level and decreasing blood pressure [7, 8]. The bioactivities of BG and B are much more than those of WG and W [6]. In previous studies of extraction methods, a variety of approaches have been developed for the extraction of useful components from* S. baicalensis*, for instance, soxhlet extraction (SE), heating reflux extraction (HRE), supercritical fluid extraction (SFE) [9], ultrasonic assisted extraction (UAE) [10], and microwave assisted extraction (MAE) [11]. Moreover, water, methanol, ethanol, and ethyl acetate are commonly used solvents for the extraction of bioactive compounds from plant materials and oriental medicine herbs (OMHs). Identification of the bioactivity compounds in* S. baicalensis* has been achieved through several methods, including thin-layer chromatography (TLC), high performance liquid chromatography (HPLC), high-speed counter-current chromatography (HSCCC), capillary electrophoresis (CE), and micellar electrokinetic capillary chromatography (MEKC) [12]. In the past few years, online screening with a HPLC postcolumn assay involving the DPPH or ABTS radical technique has been developed, allowing bioactive compounds to be spectrophotometrically monitored. Also, this method was successfully applied for screening and identifying natural bioactive compounds from complex mixtures, especially for extracts of OMHs [13, 14].

This work investigates applications of offline ABTS IC_50_ assays and online screening HPLC-ABTS assays for bioactivity screening, so that a more practical approach may be taken towards the use of online screening HPLC-ABTS assays for the rapid pinpointing of bioactivity peaks in chromatograms finds expression in experimental. Additionally, various solvent extraction techniques are compared in terms of their yields of the four major compounds: glycoside baicalin (BG), wogonoside (WG), aglycon baicalein (B), and wogonin (W) compounds in* S. baicalensis*. And, their anti-inflammatory activities were evaluated via analyzed inhibitory effect on NO and inflammatory cytokine production.

## 2. Experimental

### 2.1. Reagents and Materials

**Figure 1:**
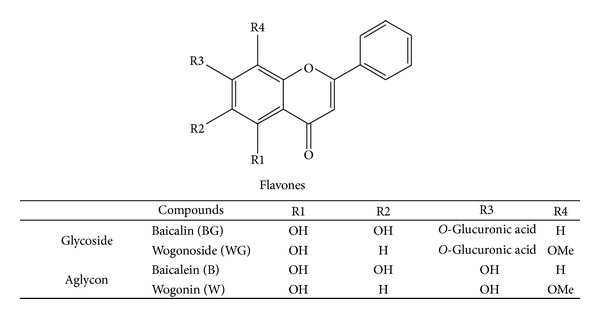
Chemical structure of baicalin, wogonoside, baicalein, and wogonin compounds in* S. baicalensis*.

The following reagents were used for radical-scavenging assays: ABTS (2,2′-azino-bis-3-ethylbenzothiazoline-6-sulfonic acid), potassium persulfate, and trifluoroacetic acid (TFA) were purchased from Sigma. Co. (USA). The standard chemicals of baicalin (BG), wogonoside (WG), baicalein (B), and wogonin (W) were obtained from Sigma. Co. (USA). The dried root of* S. baicalensis* was purchased from the Yeongcheon market (Gyeongsangbuk-do, South Korea) in March 2012. HPLC-grade ethanol, methanol, and acetonitrile were purchased from J. T. Baker (USA). The triple distilled water was filtered by a pump (Division of Millipore, Merck, Billerica, MA, USA) and filter (FH-0.2 *μ*m, Waters, Milford, MA, USA). Materials for cell culture were obtained from Lonza (Basel, Switzerland). LPS, Bovine serum albumin (BSA), and 3-(4,5-dimethylthiazol-2-yl)-2,5-diphenylthiazolium bromide (MTT) were purchased from Sigma (St. Louis, MO, USA). Antibodies for ELISA were obtained from eBioscience (San Diego, CA, USA). The chemical structures of four major flavonoids BG, B, WG, and W are shown in Figure 1.

### 2.2. Standard Sample Preparation

The high purity standard sample (higher than >95%) was prepared by dissolving 2 mg of the standard chemicals baicalin (BG), wogonoside (WG), baicalein (B), and wogonin (W) in 10 mL of methanol and adjusting the concentration to 200 ppm.

### 2.3. ABTS Sample Preparation

A 2 mM ABTS stock solution containing 3.5 mM potassium persulfate was prepared and was kept in the dark at room temperature for 16 h to allow the completion of radical generation and was then diluted with water (1 : 29, v/v).

### 2.4. Solvent Extraction

5 g samples of the dry powder from the* S. baicalensis* containing the four major flavonoids, BG, WG, B, and W, were loaded in 100 mL of pure 100% water, 100% ethanol, and 80%, 70%, and 60% aqueous ethanol solution 100 mL by dipping method for 4 h at 25°C. Each extract was filtered, concentrated under vacuum using rota-evaporator, and refrigerated for 48 h. Then the samples were frozen dry and the total extraction yield was calculated. Each sample was filtered through a 0.2 *μ*m membrane filter prior to offline ABTS assay and online screening HPLC-ABTS analysis.

### 2.5. Extraction Yield

Each experiment was performed in two replicates and the data were subjected to calculations of means ± SD. The extract sample was expressed as a percentage of the weight. The extraction yield was measured using (1). Also, samples were prepared by dissolving 100 mg of the extracted solution in 2 mL of methanol and adjusting the concentration to 50 mg/mL. The solutions were applied by ultracentrifuge for 10 min at 10,000 rpm. These solutions were filtered through a 0.2 *μ*m membrane filter prior to analysis. Consider the following:

(1) Extraction  yield(%)=Extracts  dry  weightSample  dry  weight×100.

### 2.6. Offline ABTS Assay for Bioactivity Evaluation

The ABTS radical cation method [15] was modified to evaluate the free radical-scavenging effect of* S. baicalensis* extracts. The ABTS reagent was prepared by mixing 5 mL of 7 mM ABTS with 88 *μ*L of 140 mM potassium persulfate. The mixture was then kept in the dark at room temperature for 16 h to allow free radical generation and was then diluted with water (1 : 44, v/v). To determine the scavenging activity, 100 *μ*L ABTS reagent was mixed with 100 *μ*L of sample in a 96-well microplate and was incubated room temperature for 6 min. After incubation, the absorbance was measured 734 nm using an ELISA reader (TECAN, Gröding, Austria), and 100% methanol was used as a control. The ABTS scavenging effect was measured using the following formula:

(2) Radical  scavenging(%)=(A)control−(A)sample(A)control×100.

The IC_50_ ABTS values (the concentration of sample required to inhibition 50% of ABTS radicals) were obtained through extrapolation from regression analysis. The bioactivity was evaluated based on this IC_50_ value.

### 2.7. Online Screening HPLC-ABTS Assay Analysis

**Figure 2:**
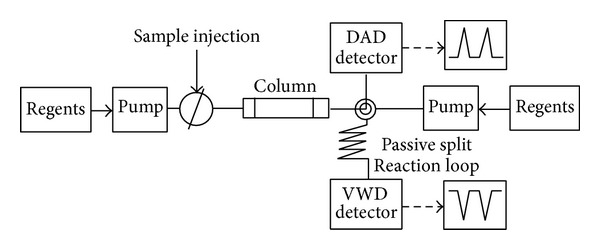
Schematic of online screening HPLC-ABTS system.

The online radical-scavenging activity of* S. baicalensis* was determined using the ABTS assay modified the methods used by Stewart et al. [16]. A 2 mM ABTS stock solution containing 3.5 mM potassium persulphate was prepared and was kept in the dark at room temperature for 16 h to allow the completion of radical generation and was then diluted with water (1 : 29, v/v).* S. baicalensis* extract was injected into a Dionex Ultimate 3000 HPLC system (Thermo scientific). The chromatographic columns used in this experiment are commercially available; this is obtained from RS-tech (0.46 × 25 cm, 5 *μ*m, C_18_, Daejeon, Korea). The injection volume was 10 *μ*L, and the flow rate of the mobile phase was 1.0 mL/min. The wavelength of the UV detector was fixed at 275 nm. The compositions of the mobile phases were A, water/trifluoroacetic acid = 99.9/0.1, vol% and B, acetonitrile 100%. The run time was 80 min and the solvent program was the linear gradient method (90 : 10-60 : 40, A : B vol%).  Figure 2 is a schematic showing the online coupling of HPLC to a DAD (Diode Array Detector) and the continuous flow ABTS assay. Online HPLC then arrived at a “*T*” piece, where ABTS was added. The ABTS flow rate was 0.5 mL/min, delivered by a Dionex Ultimate 3000 Pump. After mixing through a 1 mL loop which was maintained at 40°C, the absorbance was measured by a VIS detector at 734 nm. Data were analyzed using Chromeleon 7 software.

### 2.8. Cell Culture and Drug Treatment

RAW 264.7 cells were obtained from Korea Cell Line Bank (Seoul, Korea) and grown in RPMI 1640 medium containing 10% FBS and 100 U/mL of antibiotics sulfate. The cells were incubated in humidified 5% CO_2_ atmosphere at 37°C. To stimulate the cells, the medium was changed with fresh RPMI 1640 medium and LPS (200 ng/mL) [17, 18] was added in the presence or absence of four compounds (1, 3, 5, and 10 *μ*M) for 24 h.

### 2.9. Cell Viability Assay

Cytotoxicity was analyzed using a cell counting kit (CCK, Dojindo, Japan). Four compounds were added to the cells and incubated for 24 h at 37°C with 5% CO_2_. CCK solutions were added to each well and the cells were incubated for another 1 h. Then the optical density was read at 450 nm using an ELISA reader (Infinite M200, Tecan, Männedorf, Switzerland).

### 2.10. Measurement of NO Production

NO production was analyzed by measuring the nitrite in the supernatants of cultured macrophage cells. The cells were pretreated with five compounds and stimulated with LPS for 24 h. The supernatant was mixed with a same volume of Griess reagent (1% sulfanilamide, 0.1% naphthylethylenediamine dihydrochloride, and 2.5% phosphoric acid) and incubated at room temperature (RT) for 5 min [17]. The absorbance at 570 nm was read.

### 2.11. Determination of TNF-α, IL-6, and IL-1*β* Cytokine Production

Cells were seeded at a density of 5 × 10^5^ cells/mL in 24-well culture plates and pretreated with various concentrations of four compounds for 30 min before LPS stimulation. ELISA plates (Nunc, Roskilde, Denmark) were coated overnight at 4°C with capture antibody diluted in coating buffer (0.1 M carbonate, pH 9.5) and then washed five times with phosphate-buffered saline (PBS) containing 0.05% Tween 20. The nonspecific protein-binding sites were blocked with assay diluent buffer (PBS containing 10% FBS, pH 7.0) for more than 1 hour. Promptly, samples and standards were added to the wells. After 2 hours of incubation at RT or overnight at 4°C, the working detector solution (biotinylated detection antibody and streptavidin-HRP reagent) was added and incubated for 1 hour. Subsequently, substrate solution (tetramethylbenzidine) was added to the wells and incubated for 30 min in darkness before the reaction was stopped with stop solution (NH_3_PO_4_). The optical density was read at 450 nm [17].

### 2.12. Statistical Analysis

The results are expressed as mean ± SD values for the number of experiments. Statistical significance was compared each treated group with the control and determined by Student's *t*-tests. Each experiment was repeated at least three times to yield comparable results. Values with *P* < 0.01 and *P* < 0.001 were considered significant.

## 3. Result and Discussion

### 3.1. Extraction from* S. baicalensis*

Components from OMHs can be extracted using various extraction methods, and the extraction efficiency and component contents vary according to the extraction method. This study investigated the extraction efficiency, composition, and bioactivity of components from* S. baicalensis* using various solvent extractions. Different solvents used for the optimization of the extraction of major flavone compounds from* S. baicalensis *were water, ethanol, and 80%, 70%, and 60% aqueous ethanol. Increasing ethanol percentage decreased the yield of extracted bioactive compounds. The extraction efficiencies obtained using 60%, 70%, and 80% aqueous ethanol are 43.05%, 33.10, and 23%, respectively. Moreover, the yield and bioactivity (radical scavenging) obtained using pure water and ethanol extractions were lower than those obtained using mixed aqueous ethanol solvents (Table 1).

#### 3.1.1. Offline ABTS Assay

**Figure 3:**
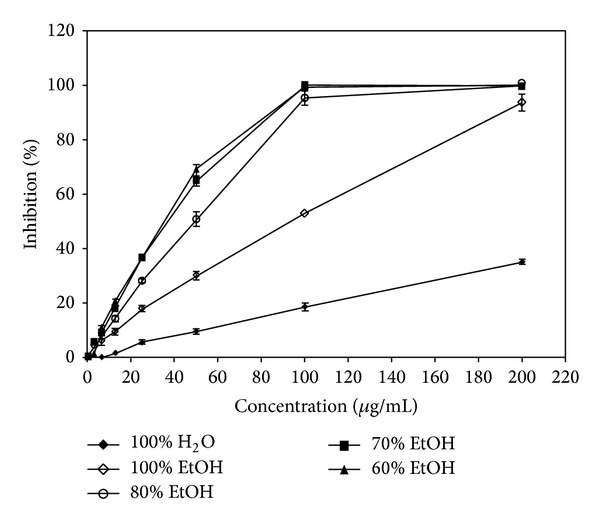
Free radical-scavenging activity of* S*.* baicalensis *extracts by ABTS assay. (◆) Water, (*⋄*) 100% EtOH, (○) 80% EtOH, (■) 70% EtOH, and (▲) 60% EtOH, results are mean ± S.D (*n* = 3).

ABTS is one of the compounds that have a proton free radical, with a characteristic absorption, which decreases significantly upon exposure to proton radical scavengers. It is well accepted that the ABTS radical scavenging by bioactivity is attributable to their hydrogen-donating ability. Accordingly, as shown in Figure 3, the ABTS radical-scavenging activity of 100% water, 100% ethanol, and 80%, 70%, and 60% aqueous ethanol, extract from* S. baicalensis* was shown to occur in a dose-dependent manner. Of these, the 60% aqueous ethanol extract showed the strongest activity. Overall, the aqueous ethanol extracts showed better inhibitory activity against the ABTS radical than water extracts. The concentration required to inhibit 50% radical-scavenging effect (IC_50_) was determined through testing a series of concentrations. The IC_50_ values of 100% water, 100% ethanol, and 80%, 70%, and 60% aqueous ethanol extracts were 302.17, 80.69, 44.87, 39.22, and 39.22 *μ*g/mL, respectively. The study of Park et al. showed that the DPPH IC_50_ value of* S. baicalensis* crude extract was 290 *μ*g/mL, which is similar to the result from a water extract in this study [19]. The figure is higher than that of this study, but there is a significant difference between two experimental methods. Consequently, this study shows that 60% aqueous ethanol has the highest bioactivity, while the 100% water extract has the lowest value. Thus, it is considered that using a water-ethanol mixture extract is suitable for water-ethanol mixture extract is higher than that of water or ethanol extract.

#### 3.1.2. Online HPLC-ABTS Assay Analysis

**Figure 4:**
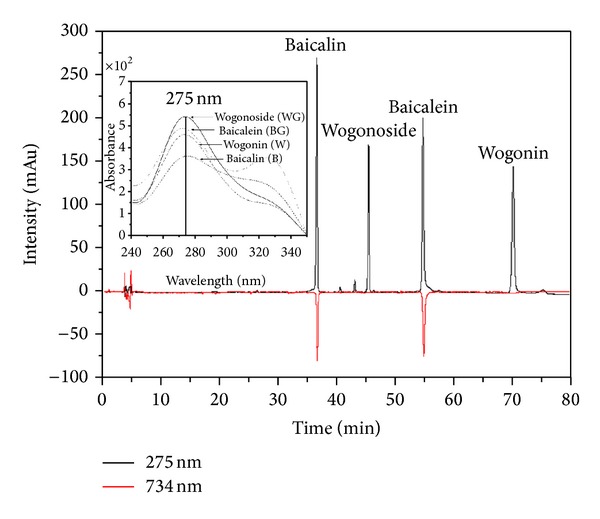
Chromatogram of online screening HPLC-ABTS radical scavenging of standard chemical compounds.

**Figure 5:**
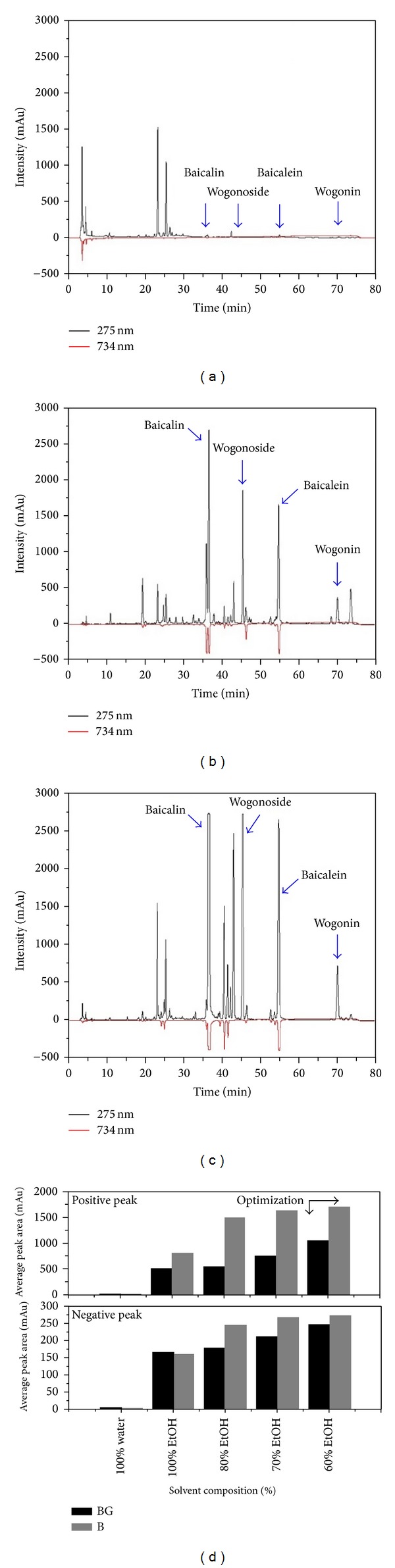
Chromatogram and column symbol of online screening HPLC profile of* S. baicalensis* extracts. Upper profile: UV signal at 275 nm, lower profile: ABTS reduction signal at 734 nm, (a) 100% water, (b) 100% EtOH, (c) 60% EtOH, and (d) negative and positive average peak area.

The HPLC separated analyses react after column with the ABTS and the reduction is detected as a negative peak by a VIS absorbance detector at 734 nm. As the ABTS radical is much more water soluble than DPPH, the ABTS assay is more widely used for evaluation of water bioactivities. Combined UV (positive signals) and ABTS quenching (negative signals) chromatograms of the different* S. baicalensis* extracts and standard chemical (200 ppm) are presented in Figures 4 and 5. Several eluted flavonoids in the extract were detected, including BG, WG, B, and W giving a positive signal on the UV detector (275 nm). Among them, the others showed hydrogen-donating capacity (negative peak) towards the ABTS radical at the applied concentration. These results revealed that the method can be applied for a quick screening of bioactivity, or more precisely, of radical-scavenging activity of compounds. In this case, BG and B were determined chromatographically, confirming their bioactivity though WG or W was not detected. This means that BG and B have the high bioactivity, whereas WG and W have the low bioactivity. In addition, Gao et al. [6] elicited similar results using a DPPH assay method. It shows the details of an online HPLC-ABTS assay system which analyzes the extracts from solvents (Figures 5(a)–5(d)). The retention time (*R*_*t*_) of glycoside BG (*R*_*t*_: 54.74~54.87 min) and WG (*R*_*t*_: 45.47~45.53 min) and aglycone B (*R*_*t*_: 36.18~36.68 min) and W (*R*_*t*_: 70.03~70.18 min) was reported by this work. The 60% aqueous ethanol extract resulted in the greatest total extraction amount (BG: 41.46, WG: 71.14, B: 62.26, and W: 10.13 mg) were the total extraction amount highest them 70% aqueous ethanol extract (BG: 22.70, WG: 54.40, B: 45.40, and W: 7.39 mg), 80% aqueous ethanol extract (BG: 11.42, WG: 38.08, B: 28.79, and W: 4.99 mg), 100% ethanol extract (BG: 0.09, WG: 1.21, B: 1.32, and W: 0.24 mg), and 100% water extract (BG: 0.21, WG: 0.01, B: 0.13, and W: 0.04 mg). Bioactivity of the 60% aqueous ethanol extract (BG: 246.00, B: 272.92 mAU) was the highest of all, as compared to the 70% aqueous ethanol extract (BG: 211.85, B: 267.07 mAU), 80% aqueous ethanol extract (BG: 179.00, B: 244.52 mAU), 100% ethanol extract (BG: 166.45, B: 161.15 mAU), and 100% water extract (BG: 6.01, B: 2.76 mAU). The bioactivity appears to be approximately proportional to the concentration of BG and B contained in the extracts. Bioactivity was not detected for WG and W, and therefore they should not exert any effect of bioactivity (radical-scavenging activity) (Table 2). Moreover, most research has focused on the transformation of BG and WG to improve the yield of B and W [20]. The major effective flavones BG and B were reported to be bioactive components [21]. Also, glycosides BG and WG were the most abundant components and have antiallergic, anti-inflammatory, anti-HIV, antioxidant, and free radical-scavenging effects [22]. This work confirms the feasibility of assessing the bioactivity of specific phytochemicals, using an online screening HPLC-ABTS assay method. It was successfully applied for screening and identifying natural bioactivity from OMHs and natural substance complex mixtures.

### 3.2. Anti-Inflammatory Activities Screening

#### 3.2.1. Effect of Four Compounds on RAW 264.7 Cell Viability

**Figure 6:**
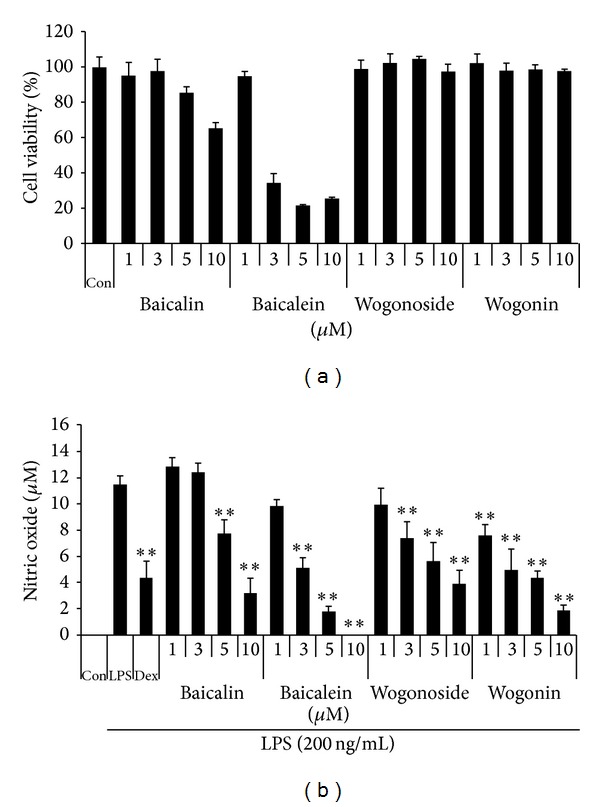
Effect of four compounds on (a) cell viability and LPS-induced (b) NO production in RAW 264.7 cells. RAW 264.7 cells were pretreated with four compounds for 30 min before incubation with LPS for 24 h. (a) Cytotoxicity was evaluated by a CCK. (b) The culture supernatant was analyzed for nitrite production. As a control, the cells were incubated with vehicle alone. Data shows mean ± SE values of triplicate determination from independent experiments. *P < 0.01 and **P < 0.001 were calculated from comparing with LPS-stimulation value.

We evaluated the cytotoxicity of four compounds using a CCK to determine the optimal concentration that would be effective for anti-inflammation with minimum toxicity. As shown in Figure 6(a), baicalin shows little toxicity at concentration of 10 *μ*M. Also, baicalein contains strong toxicity on macrophage viability at 3 *μ*M or more. Wogonoside and wogonin did not affect cell viability up to 10 *μ*M, indicating two compounds are not toxic to cells.

#### 3.2.2. Effect of Four Compounds on NO Production in LPS-Stimulated RAW 264.7 Macrophages

We evaluated the effects of four compounds on NO secretion in LPS-stimulated RAW 264.7 cells. The cells were pretreated with four compounds at various concentrations prior to LPS stimulation and NO production was measured. As a positive control, we employed 10 *μ*M dexamethasone, which is widely employed as an anti-inflammatory agent. As shown in Figure 6(b), baicalin shows slightly inhibitory effect on NO secretion upon LPS stimulation at 5 *μ*M. Baicalein repressed NO secretion at concentrations of 3 *μ*M or more. However, this inhibitory effect was related with strong cytotoxicity of baicalein [18]. Wogonoside and wogonin were strongly inhibited NO production in a dose-dependent manner with statistical significance.

#### 3.2.3. Effect of Four Compounds on LPS-Induced Inflammatory Cytokines Production

**Figure 7:**
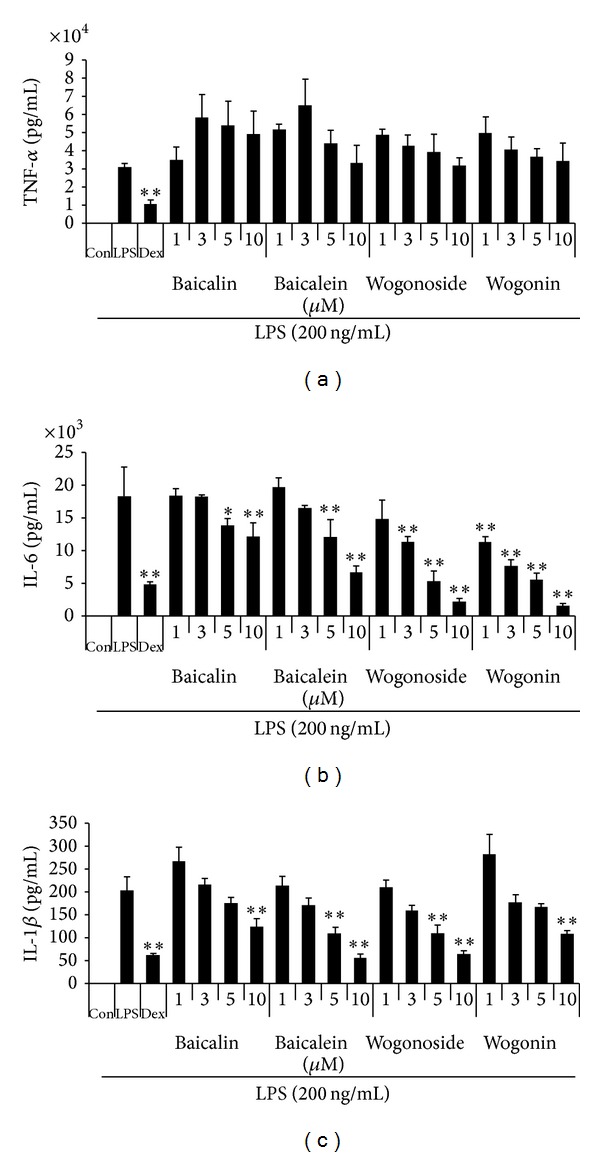
Effect of four compounds on the production of (a) TNF-α, (b) IL-6, and (c) IL-1*β* cytokine in macrophages. Cells were pretreated with four compounds for 30 min before being incubated with LPS for 24 h. Production of cytokines was measured by ELISA. Data shows mean ± SE values of duplicate determinations from three independent experiments. *P < 0.01 and **P < 0.001 were calculated from comparing with LPS-stimulation value.

Next, we investigated the inhibitory effect of four compounds on the production of inflammatory cytokines, which is another parameter of inflammation. In this study, we examined the effect of four compounds on TNF-α, IL-6, and IL-1*β* cytokine production. As shown in Figure 7(a), all compounds did not inhibit TNF-α production at all concentrations. As shown in Figure 7(b), wogonoside and wogonin were significantly inhibited IL-6 cytokine secretion at concentrations of 3 *μ*M or more with statistical significance. Consistent with IL-6 results, wogonoside and wogonin showed inhibitory effect on IL-1*β* cytokine production in a dose-dependent fashion (Figure 7(c)).

## 4. Conclusions

This study provides the comparison of free radical scavengers in the extracts of* S. baicalensis* by an offline ABTS assay and an online screening HPLC-ABTS assay. The results showed the effect of solvent composition on total extraction yield from* S. baicalensis*. Based on our investigations, total extract yield and bioactivity decreased with ethanol increasing in the solvent mixture. In addition, the bioactivities of BG and B were determined to be much greater than those of WG or W. The yield (43.05%) and IC_50_ (34.04 *μ*g/mL) determined through ABTS assay of the 60% aqueous ethanol extract were the most satisfactory of all solvent solutions tested. And, this confirms the feasibility of assessing the bioactivity of specific phytochemicals using the online screening HPLC-ABTS assay. There was a very small margin of error between the results of the offline ABTS assay and those of the online screening HPLC-ABTS assay. Moreover, their anti-inflammatory activities were evaluated via analyzed inhibitory effect on NO and inflammatory cytokine production. Furthermore, WG and W exhibited the strong inhibitory effects on inflammatory mediator production including NO, IL-6, and IL-1*β* in LPS-stimulated RAW 264.7 macrophages. In conclusion, these compounds could be developed as a new anti-inflammatory therapeutic agent without cytotoxicity. These results will be compiled as a database, for use in investigating the constituents of natural products and the resources of pharmaceutical, nutrition, and cosmetic products.

## Figures and Tables

**Table 1:** Yield of extraction solvent composition and bioactivity of the *S. baicalensis *extracts by free radical-scavenging activity ABTS IC_50_ assay.

Extraction solvent	Extraction yield (%)	IC_50 _(*μ*g/mL)
Water 100%	18.36 ± 0.25^a^	302.17 ± 37.40^b^
EtOH 100%	1.97 ± 0.01	80.69 ± 2.10
EtOH 80%	23.00 ± 0.90	44.87 ± 4.76
EtOH 70%	33.10 ± 0.10	39.22 ± 2.74
EtOH 60%	43.05 ± 0.47	34.04 ± 3.22

^a^Each value in mean ± SD (*n* = 2); ^b^each value in mean ± SD (*n* = 3).

**Table 2:** Comparison of extracts efficiency by online screening HPLC-ABTS in positive and negative peak area.

Extraction solvent (%)	Compounds	*R*_*t*_ (min)	Positive peak					Negative peak		
			Average peak area (mAU)	Standard deviation (SD±)	RSD^a^ (%)	Yield (%)	Total extraction amount (mg)	Average peak area (mAU)	Standard deviation (SD±)	RSD (%)
100% Water	BG	54.76	12.80	2.54	19.86	1.07	0.21	6.01	0.01	0.08
	WG	45.53	0.20	0.22	114.26	0.01	0.01	ND^b^	—	—
	B	36.42	8.71	10.32	118.52	0.96	0.13	2.76	3.91	141.42
	W	70.11	2.93	1.32	45.18	0.23	0.04	ND	—	—

100% Ethanol	BG	54.77	505.72	26.58	5.26	12.84	0.90	166.45	1.79	1.08
	WG	45.48	468.99	24.32	5.19	11.91	1.21	ND	—	—
	B	36.18	803.00	126.99	15.81	20.15	1.32	161.15	15.61	9.69
	W	70.18	164.19	13.56	8.26	4.16	0.24	ND	—	—

80% Aqueous ethanol	BG	54.74	545.35	31.60	5.80	8.41	11.42	179.00	13.59	7.59
	WG	45.47	1258.84	232.65	18.48	19.10	38.08	ND	—	—
	B	36.60	1492.57	372.90	24.98	22.46	28.79	244.52	49.46	20.23
	W	70.03	292.71	16.91	5.78	4.51	4.99	ND	—	—

70% Aqueous ethanol	BG	54.76	753.16	26.72	3.55	11.34	22.70	211.85	21.91	10.34
	WG	45.50	1249.30	208.61	16.70	18.53	54.40	ND	—	—
	B	36.68	1634.98	384.09	23.49	24.07	45.40	267.07	53.40	20.00
	W	70.06	300.91	16.19	5.38	4.52	7.39	ND	—	—

60% Aqueous ethanol	BG	54.78	1049.88	34.23	3.26	15.12	41.46	246.00	29.69	12.07
	WG	45.51	1246.60	104.08	8.35	17.78	71.14	ND	—	—
	B	36.59	1710.77	194.19	11.35	24.26	62.26	272.92	56.03	20.53
	W	70.05	314.99	6.94	2.20	4.55	10.13	ND	—	—

^a^RSD: relative standard deviation; ^b^ND: not detected.
